# Network analysis and experimental approach to investigate the potential therapeutic mechanism of zishen yutai pills on premature ovarian insufficiency

**DOI:** 10.1016/j.heliyon.2023.e20025

**Published:** 2023-09-17

**Authors:** Zifan Song, Kuangyu Song, Hongru Zhao, Yuanqiao He, Jia Hu

**Affiliations:** a Department of Obstetrics and Gynecology, The First Affiliated Hospital of Nanchang University, Nanchang, Jiangxi, China; b Department of Physiology, Basic Medical College, Nanchang University, Nanchang, Jiangxi, 330006, PR China; c Department of Microbiology, School of Medicine, Nanchang University, Nanchang, Jiangxi, 330006, PR China; d Center of Laboratory Animal Science, Nanchang University, Nanchang, Jiangxi, China; e Jiangxi Province Key Laboratory of Laboratory Animal, Nanchang, Jiangxi, 330031, PR China; f Nanchang Royo Biotech Co,. Ltd, Nanchang, Jiangxi, China

**Keywords:** Zishen yutai pills, Premature ovarian failure, Transcriptomics, Network pharmacology

## Abstract

**Background:**

As society continues to develop, women are more at risk of gonadotoxic substance exposure. Consequently, the incidence of premature ovarian insufficiency (POI) has increased significantly in the past decades. Hormone replacement therapy (HRT) is recommended as the standard treatment to relieve hypoestrogenic symptoms; however, its potential side effects and contraindications have drawn widespread controversy and concern. As such, the Chinese medicine Zishen Yutai Pill (ZSYTP) commonly used for treating miscarriage and menoxenia, is a highly promising alternative drug candidate against POI, however its therapeutic mechanism has not been completely elucidated.

**Objective:**

To systematically analyze the potential therapeutic targets of ZSYTP on POI, we combined network pharmacology analysis and molecular docking to predict critical target genes, with experimental validation on POI murine models.

**Methods:**

The active compounds of ZSYTP were collected from three online databases, and the candidate targets were predicted based on the chemical structure. The POI-related targets were obtained from four databases. A PPI network was constructed to find the key target genes between ZSYTP and POI, while GO and KEGG enrichment analyses were employed to study the mechanism of ZSYTP against POI. The binding capability of the key co-targets with active components was examined by molecular docking. We used a cyclophosphamide (CTX)-inducible POI mouse model to verify our predictions by histopathological observation, immunohistochemical staining (caspase-3, TUNEL assay), hormone determination (FSH, AMH) and ribonucleic acid sequencing (RNA Seq). Progynova was also used to study the difference between ZSYTP and HRT.

**Result:**

We identified 21 target genes as the hub between ZSYTP and POI. The GO and KEGG analyses revealed that the molecular mechanism of ZSYTP against POI were mainly based on the regulation of gene and protein expression. A variety of signaling pathways may be involved in the treatment of ZSYTP against POI, especially PI3K-AKT, HIF-1 and the AGE-RAGE cascades. Docking simulation showed that G1, C1, SR5, and F1 had relatively lower binding energy. *In vivo*, ZSYTP significantly reversed CTX-induced ovarian damage in follicle number, hormone level and apoptosis, with an overall improved therapeutic effect compared to Progynova. Results from RNA-Seq revealed that the PI3K-AKT, Hippo, AGE-RAGE, and Rap1 signaling pathways and regulation of inflammation, immune response, and lipid metabolism may mediate the protective effects of ZSYTP against POI, which is different than Progynova's mechanism of action.

**Conclusions:**

Collectively, this study indicates that ZSYTP could be a highly promising alternative as a non-HRT-based therapy for POI. Its mechanism involves multiple signaling pathways, alleviating ovarian apoptosis and recovering AMH and FSH level. However, the discrepancy between different research techniques highlight the necessity of further experimental verification from other aspects such as translation and posttranslational modification.

## 1 Introduction

The reproductive decline in women is an inevitable biological process, often accompanied by emotional distress and various diseases. Thus, the progression of this natural phenomenon, namely premature ovarian insufficiency (POI), causes subfertility in women of reproductive age and lowers their quality of life. POI is defined as a pathological state wherein ovarian hypofunction occurs before age 40, and mainly characterized by abnormal menstruation, hypoestrogenic and hypergonadotropic conditions. One of the key symptoms in the pathogenesis of POI is follicular atresia that is primarily mediated by upregulation of apoptosis. Approximately 1% of women aged under 40 years and 0.1% of women aged under 30 years suffer from POI [1]. Although the etiology of POI remains poorly defined, many studies have suggested that multiple factors, such as genetic and metabolic disorders, may contribute to the onset of POI. Iatrogenic procedures necessary for the treatment of cancer, including ovarian surgeries, radiotherapy, and chemotherapy, have become important causes of secondary POI [[2], [3], [4]]. Although hormone replacement therapy (HRT) is strongly recommended in clinics as the first-line treatment of POI, it can cause significant side effects due to the use of exogenous steroidal hormones [5,6]. To address this issue, many innovative treatment strategies have been developed, such as stem cell and exosome therapies; however, these new techniques are still in the experimental stage [7]. Hence, it is necessary to find novel and safe therapeutic alternatives to treat POI.

Zishen Yutai Pill (ZSYTP), consisting of 15 Chinese medicinal compounds, is a patented Chinese prescription now widely used to treat a diverse range of reproductive disorders, including absent and irregular menstruation [8]. In the past decade, ZSYTP has been shown to significantly ameliorate the blood supply of female reproductive organs and stimulate follicular development and progestin secretion, suggesting a favorable effect of promoting ovarian function [9,10]. In addition, ZSYTP facilitates the recovery from POI either when used singularly or in combination with other medicines [[11], [12], [13]]. The safety of ZSYTP is likewise reliable, showing no significant toxicity or adverse side effects [[14], [15], [16], [17]]. Therefore, this drug may be a promising alternative to HRT. However, the underlying mechanism of ZSYTP in treating POI remains unclear. Unlike synthetic drugs, the regulatory mechanism of Chinese medicinal drugs consists of multiple components and targets. As such, to study Chinese medicinal compounds, we ought to adopt a novel integrated research system that combines the holism concept of traditional Chinese medicine (TCM) with multi-disciplinary approaches.

**Fig. 1:**
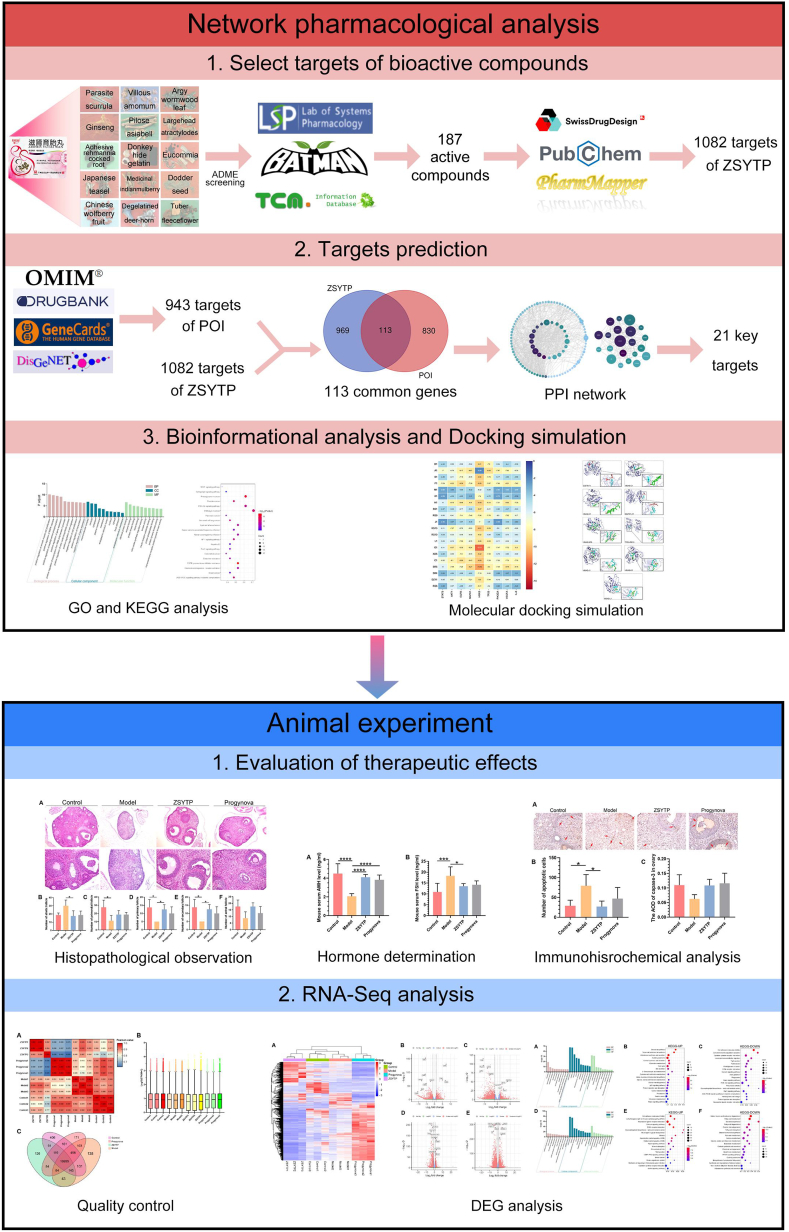
Flow chart of the study.

In this study, we used network pharmacology analysis to predict the potential therapeutic targets of ZSYTP on POI. Animal experiments, including histopathology and immunochemistry, were also performed to verify our *in silico* predictions. In addition, to more confidently evaluate the accuracy of the network predictions, we employed RNA sequencing (RNA-Seq) to obtain a comprehensive transcriptome profiling. The overall workflow of this study is provided in Fig. 1.

## 2 Materials and methods

### 2.1 Data preparation

#### 2.1.1 Screening for active components and targets of ZSYTP

All 15 traditional Chinese herbs used to prepare ZSYTP were mainly collected from the TCMSP (http://tcmspw.com/tcmsp.php, accessed September 2022) database [18], as well as the BATMAN-TCM (http://bionet.ncpsb.org/batman-tcm/, accessed September 2022) and TCMID (http://www.bidd.group/TCMID/index.html, accessed September 2022) databases to identify any potential components of ingredients missing from TCMSP [19,20]. To improve precision, we also queried the CNKI, WanFang, VIP, and Pubmed databases for any other crucial ingredients.

After collecting candidate components of ZSYTP and deduplicating, their chemical information, including canonical SMILES, “mol2” files, and 2D molecular structures, were obtained from TCMSP, PubChem, and TCMID and then input into SwissTargetPrediction (http://www.swisstargetprediction.ch/, accessed September 2022) and PharmMapper (http://www.lilab-ecust.cn/pharmmapper/, accessed September 2022) to predict potential targets based on the similarity principle [21,22]. Finally, the gene symbols and protein names were standardized via the UniProt database (https://www.uniprot.org/, accessed September 2022) [23].

Our screening criteria were as follows: (1) In TCMSP: oral bioavailability (OB) > 30%; drug-likeness (DL) > 0.18. (2) In BATMAN: Score cut-off >20; *p* < 0.05. (3) In SwissTargetPrediction: Probability >0. (4) In PharmMapper: Normalized Fit Score >0.8.

#### 2.1.2 Selection of common targets between ZSYTP and POI

According to the European Society of Human Reproduction and Embryology (ESHRE) guidelines issued in 2016, “premature ovarian insufficiency” was suggested as the official terminology [24]. It was defined as a clinical syndrome with declined ovarian function in women before the age of 40 years. However, various names and definitions were used in the past, such as premature ovarian failure (POF) and primary ovarian insufficiency. To ensure accuracy, we decided to adopt the following academic terms, “premature ovarian insufficiency” and “premature ovarian failure,” as keywords to acquire POI-related genes. As highlighted by the Chinese expert consensus on clinical diagnosis and treatment of premature ovarian insufficiency in 2017, POI and POF can be regarded as two different stages of one disease [25]. Most importantly, they share the following critical, clinical, and biochemical characteristics: (1) an elevated follicle-stimulating hormone (FSH) level >25 IU/L, (2) abnormal menstruation (mostly oligomenorrhea or amenorrhea), and (3) disease onset before the age of 40 years.

Four diseases databases were used for gathering POI targets: GeneCards (https://www.genecards.org/, accessed September 2022), OMIM (https://www.omim.org, accessed September 2022), Drugbank (https://go.drugbank.com/, accessed September 2022), and Disgenet (https://www.disgenet.org/home/, accessed September 2022) [[26], [27], [28], [29]]. The filter setup was as follows: (1) In DisGeNET: Score_gda >0.1. (2) In Genecards: Relevance score ≥ median, performed twice. After aggregating and deduplicating, these data were stored for further analysis.

To visualize the overlapping targets between ZSYTP and POI, a Venn diagram was generated through the Bioinformatics Evolutionary Genomics website (http://bioinformatics.psb.ugent.be/webtools/Venn/, accessed September 2022). The detailed information on intersected genes was stored in Excel format for further network construction.

### 2.2 Network construction and bioinformatics analysis

The prediction of protein interaction is vital for identifying the core regulatory genes. Thus, all the common genes were uploaded into the STRING database to construct a protein–protein interaction (PPI) network using the multiple proteins option. Several filters were set as the cut-off criteria: (1) Organisms: *Homo sapiens*. (2) Confidence score >0.7. The result was downloaded in TSV format and was input into Cytoscape software (version 3.8.0) to perform network analysis. The key common genes were filtered out based on the calculations of CentiScaPe 2.2 in Cytoscape. The filter parameters were as follows: (1) Degree >12.718446601941748. (2) Betweenness >159.2233009708738. (3) Closeness >0.003982711176070545. The qualified genes were used for subsequent bioinformatic analysis.

An ingredient-target network was also constructed using Cytoscape. Nodes represented ingredients of each herbal component of ZSYTP and their target genes, while edges showed the relationship among different nodes. The color shade and node size indicated the values of degree and betweenness, respectively.

To better understand the potential mechanism of key common targets, Gene Ontology [30] (GO; http://www.geneontology.org/) and Kyoto Encyclopedia of Genes and Genomes (KEGG) (https://www.kegg.jp/) pathway enrichment was performed using the DAVID database (https://david.ncifcrf.gov/home.jsp, accessed September 2022) [31]. The top 10 terms of GO analysis with *p* < 0.05 were selected. GraphPad Prism (version 8.0) and Rstudio (version 3.6.3) were used to display the results.

### 2.3 Molecular docking

To verify the binding of the chemical components and targets, molecular docking was carried out on nine key co-targets, and 20 active components of the highest degree in the ingredient-target network were selected for docking simulations (Table 1). The 3D structures of target proteins representing docking receptors were downloaded from the PDB database (http://www.rcsb.org/, accessed September 2022). The PyMol software (version 2.x) was then used to remove the ligands and water from the protein backbone. A Mol2 file of small molecule compounds was acquired from the TCMSP database. The target proteins and small molecule ligands were imported into AutoDockTool (version 1.5.7) in PDBQT format and used to perform the docking simulation. The docking results were visualized by PyMol.

**Table 1:** List of top 20 active compounds of ZSYTP.

Abbreviation	Source	Degree	Active compound
BS1	BS	125	12-senecioyl-2E,8E,10E-atractylentriol
RS10	RS	119	Gomisin B
RS13	RS	119	suchilactone
RS9	RS	121	Girinimbin
SR4	SR	112	icosa-11,14,17-trienoic acid methyl ester
M1	GQZ, AY	229	Mandenol
A1	TSZ, RS, XD, DZ, BJT, GQZ, AY, SR	359	beta-sitosterol
SR5	SR	113	vitamin-e
D1	TSZ, SJS, DZ, GQZ, AY	525	quercetin
F1	RS, DS, GQZ, SDH, AY, SR	252	Stigmasterol
K1	DS, SR	189	methyl icosa-11,14-dienoate
RS6	RS	109	arachidonate
XD6	XD	115	Sylvestroside III_qt
H1	XD, SJS, BJT, SDH	178	sitosterol
DZ11	DZ	110	(−)-Tabernemontanine
J1	DZ, DS	120	3-beta-Hydroxymethyllenetanshiquinone
L1	DS, GQZ	118	glycitein
B1	TSZ, RS, DZ	315	kaempferol
G1	RS, DS	116	Chrysanthemaxanthin
C1	TSZ, GQZ	114	CLR

### 2.4 Verification of *in vivo* experiments

#### 2.4.1 Chemicals and reagents

The ZSYTP was provided by Guangzhou Baiyunshan Zhongyi Pharmaceutical Co., Ltd (Guangzhou, China). Cyclophosphamide (CTX) was purchased from Macklin Inc (Shanghai, China). High-performance liquid chromatography was performed for quality control [32]. According to the dosage exchange formula from humans to mice, the ratio of experimental to clinical dose was 1: 9.1 [33]. All medication was dissolved in PBS to prepare a solution of appropriate concentration: CTX 5 mg/ml, ZSYTP 0.15 g/ml, Progynova 0.01 mg/ml. The anti-caspase-3 antibody and terminal deoxynucleotidyl transferase dUTP nick end-labeling (TUNEL) Assay Kit-HRP-DAB were purchased from Abcam (London, UK). Chemiluminescence immunoassay (CLIA) Kit for FSH and anti-müllerian hormone (AMH) were both provided by Cloud-Clone Corp. Wuhan (Wuhan, China). All other chemicals and reagents were obtained from Nanchang Royo Biotech Co., Ltd. (Nanchang, China).

#### 2.4.2 Animals and treatment

**Fig. 2:**
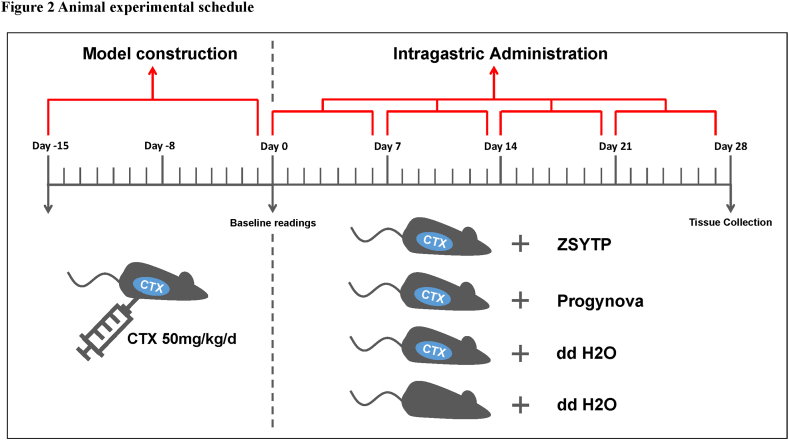
Animal experimental schedule.

Female C57BL/6 mice (8-week-old, 25–30 g) were purchased from Zhejiang Vital River Laboratory Animal Technology Co., Ltd. (Jiaxing, China) and were acclimated without any experimental manipulation for 1 week. The mice were randomly divided into four groups, each containing ten mice. To build the POI model, CTX (50 mg/kg/day) was given to mice by intraperitoneal injection for 15 days. The control group did not receive any treatment. The ZSYTP (2.25 g/kg/day) and Progynova (0.15 mg/kg/day) were administrated by gavage after exposure to CTX. The administration lasted for a total of four weeks, with a 1-day interval after every six days. At week 4, all mice were euthanized to collect serum samples through orbital sinus puncture and harvest ovaries for further analysis. The treatment schedule is shown in Fig. 2.

All animal experiments conformed to the guidelines of the Institutional Animal Care and Use Committee of Nanchang Royo Biotech Co., Ltd (IACUC Issue No: RYE2021012201).

#### 2.4.3 Histopathological observation and follicle counting

Hematoxylin and eosin staining was performed as described in our previous study [34]. The number and area of growing follicles were counted using the Image J software (NIH, USA) to evaluate ovary function. To avoid double counting, we only counted the follicles with oocyte nuclei. The mean value of each group was computed on three serial sections from three individuals.

#### 2.4.4 Immunohistochemistry and TUNEL staining

The process of immunohistochemical staining can be found in our previous study [34]. To perform TUNEL staining, slides were rehydrated in xylene and graded ethanol, then incubated with Proteinase K solution (1: 100) for 20 min. Next, H_2_O_2_ (3%, containing methanol) was applied to each specimen for 5 min to inactivate endogenous peroxidases. For equilibration, the specimens were covered with TdT Equilibration Buffer for 30 min. The TdT Labeling Reaction Mix was applied onto each specimen and co-incubated in a humidified chamber for 1.5 h at 37 °C. The Stop Buffer was added to terminate the labeling reaction after blocking for 10 min. The diluted conjugate was then applied to each specimen for 30 min in a humidified chamber. Next, the specimens were covered with DAB solution for 15 min and counterstained with hematoxylin for 3 min. The samples were then dipped in solutions of 100% ethanol and 100% xylene. A light microscope was used to count the number of TUNEL-positive cells.

#### 2.4.5 Hormone determination

The CLIA was used to measure the levels of FSH and AMH in blood according to the manufacturer's instructions. Approximately 50 μL of standards or serum samples were added into microwell plates pre-coated with anti-FSH or anti-AMH monoclonal antibody, then 50 μL of detecting solution A was added, and the plates were incubated at 37 °C for 1 h. After washing the plates three times with PBS, 100 μL detecting solution B was added and plates were incubated at 37 °C for a further 30 min. Subsequently, 100 μL substrate was added into each well after rinsing the plates five times, and the plates were incubated at 37 °C for 10 min. The hormone concentration was assessed by chemiluminescence apparatus based on the relative optical unit.

#### 2.4.6 RNA extraction, library construction, and sequencing

Mice in estrus were sacrificed under anesthesia. Their unilateral ovarian tissue was divided appropriately and transferred to a corresponding grinding tube. After dispensing 1.5 mL TRIzol lysis buffer to a 2-mL tube, the samples were grounded into a powder with liquid nitrogen and transferred into the lysis buffer. The tubes were kept at 25 °C for 5 min, then centrifuged at 12,000×*g* for 5 min at 4 °C. The supernatant was transferred to new tubes containing 300 μL chloroform/isoamyl alcohol (24:1) and mixed thoroughly, then centrifuged again for 8 min at 4 °C. The supernatant was then collected in 1.5 mL EP tubes (containing 2/3 volume of isopropyl alcohol) and gently inverted, then stored at −20 °C for 2 h.

The tubes were then centrifuged at 17,500×*g* for 25 min at 4 °C. The pellets were resuspended in 0.9 mL 75% ethanol, and the tubes were recentrifuged at 17,500×*g* for 3 min at 4 °C. The liquid was then poured off, and the tubes were centrifuged briefly, after which, we used sterile pipette tips to remove any residual liquid. Following air-drying, the precipitates were finally dissolved in 20–200 μL DEPC-H_2_O.

Sample QC was performed before preparing the sequencing library. The quality of the extracted RNA samples was evaluated by Fragment Analyzer 5200 with Standard Sensitivity RNA Analysis Kit (15 nt; DNF-471; Agilent Technology, Palo Alto, United States), including RNA concentration, 28S/18S, and RIN/RQN. Following the library construction guidelines, the samples were diluted to fit the range of RNA input concentrations.

The procedure of mRNA library preparation was as follows: First, 16 μL RNA sample was diluted to 80 μL with deionized water and denatured at 65 °C. Oligo (dT)-attached magnetic beads were used to enrich mRNA from total RNA. A fragmented reagent was added to the mRNA at a suitable temperature for a period of co-reaction, then double-stranded cDNA was synthesized using N6 primer and subjected to end-repair. A single adenine residue was added to the 3′ ends of the cDNA fragments. After ligating the cDNAs with adapters, the products were amplified by PCR and underwent heat denaturation and circularization. The single-strand circle products were replicated via rolling cycle amplification to generate DNA nanoballs (DNBs). Sufficient quality DNBs were then sent into patterned nanoarrays and sequenced on the DNBSEQ platform.

#### 2.4.7 Statistical analysis

Data from animal experiments are represented as the means ± standard deviations [35]. GraphPad Prism 8 software was used for statistical analysis and graph plotting. Differences between two groups were analyzed by Student's t-test, while data from more than two groups were compared by one-way analysis of variance (ANOVA) followed by LSD post-hoc test. A *p* < 0.05 was considered statistically significant.

## 3 Results

### 3.1 Component-target network

A total of 219 ingredients derived from the 15 herbal components of ZSYTP were collected (Supplementary Table 1). After aggregating and deduplicating, 187 components and 1082 related targets of ZSYTP were input into Cytoscape to construct a component-target network (Supplementary Table 2 and 3). The component-target network contained 1283 (1082 targets, 186 compounds, and 15 herbs) nodes and 11242 edges (Supplementary Figure 1). To display the connection more clearly, we added different geometries to nodes according to their categories. Octagons represent herbs and their ingredients. Rectangles represent common ingredients among several herbs. Rhombi represent the target genes of each ingredient. Several ingredients, such as beta-sitosterol (Degree 359), stigmasterol (Degree 252), mandenol (Degree 229), quercetin (Degree 525), and kaempferol (Degree 315), potentially play major roles in facilitating the pharmacological effects of ZSYTP. According to the same standards, CYP19A1 (Degree 116), AR (Degree 105), ESR1 (Degree 98), ESR2 (Degree 100), and other genes can be regarded as the main therapeutic targets of ZSYTP.

### 3.2 Component-POI PPI network

**Fig. 3:**
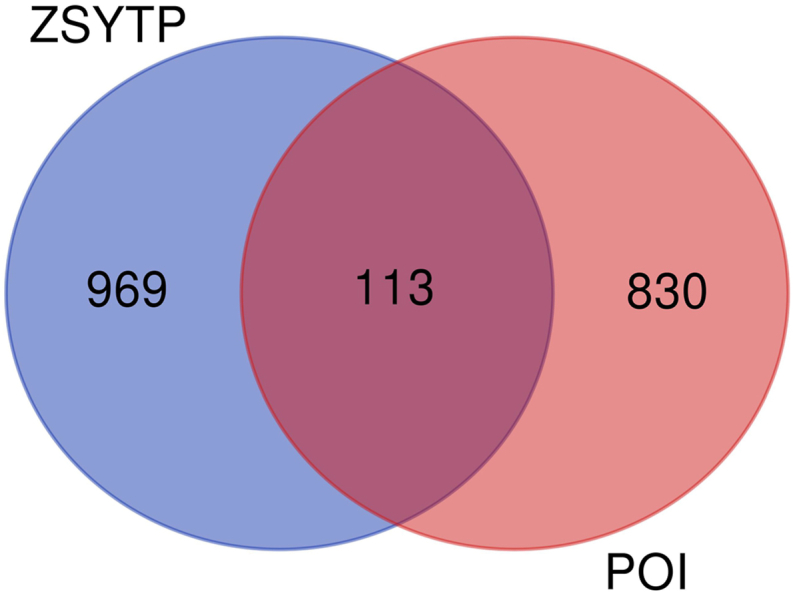
The 113 overlapping genes between ZSYTP and POI.

A total of 943 POI-related targets were acquired from four disease databases (Supplementary Table 4). A total of 1082 potential targets of ZSYTP were screened out. As shown in Figs. 3 and Figs 11 targets were located at the intersection of “drug-disease”, which may play an important role in ZSYTP against POI (more details can be found in Supplementary Table 5).

**Fig. 4:**
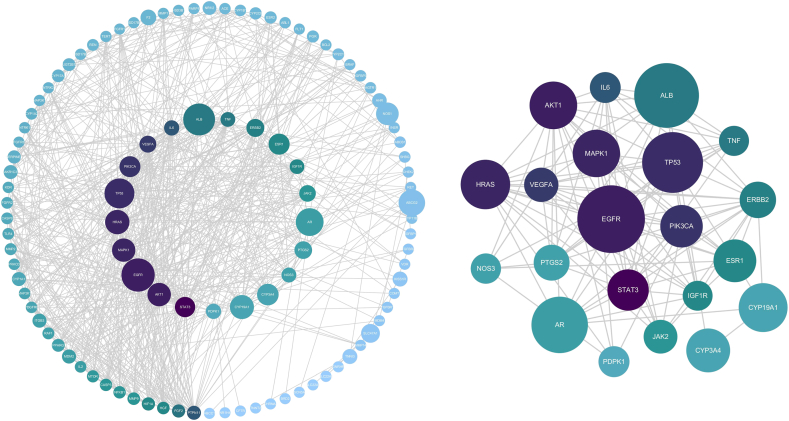
Protein interaction network of the common targets between ZSYTP and POI. Protein–protein interaction (PPI) network of 113 overlapping targets between ZSYTP and POI. The shade of the color represents the Degree value. The area of nodes represents the Betweenness value. Twenty-one key targets for ZSYTP on POI were screened based on Degree, Betweenness, and Closeness.

A component-POI PPI network was generated via the STRING website, which comprised 103 nodes (isolated nodes were excluded) and 655 edges (Fig. 4A). “Degree,” “Betweenness Centrality,” and “Closeness Centrality” were employed to measure the magnitude of the proteins in the PPI network (Supplementary Ta 6). Twenty-one targets were evaluated as the hubs between ZSYTP and POI (Fig. 4B). Among these, STAT3 (Degree 42), EGFR (Degree 38), AKT1 (Degree 38), MAPK1 (Degree 37), and HRAS (Degree 37) were the five most important targets in the core PPI network. Compared with the component-target network, these results differed substantially, stressing the need to experimentally elucidate the therapeutic effects of ZSYTP on POI.

### 3.3 GO and KEGG analysis

#### 3.3.1 GO functional enrichment analysis

**Fig. 5:**
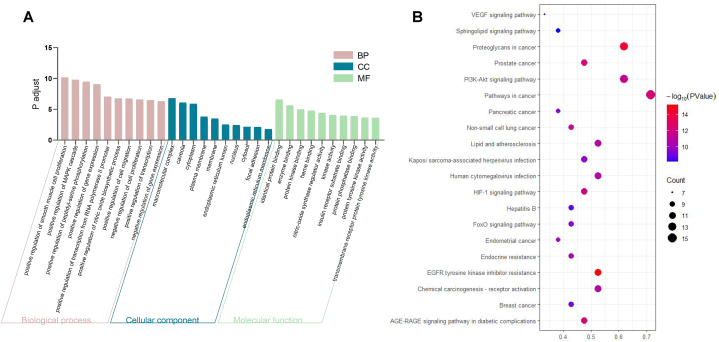
Bioinformational analysis of overlapping gene symbols related to POI. (A) Gene Ontology [30] analysis of overlapping gene symbols related to POI. A p-value <0.05 served as the significance threshold. The top 10 terms of BP, CC, and MF are shown according to their p.adjust (-log_10_ p-value). (B) Kyoto Encyclopedia of Genes and Genomes (KEGG) pathway enrichment analysis. The X-axis indicates Gene Ratio. The Y-axis shows the pathway name.

We selected the 21 key targets for GO enrichment analysis to more clearly elucidate the protective effect of ZSYTP in the body against POI. Fig. 5A displays the top 10 terms of biological process (BP), molecular function (MF), and cellular component [27]. More details of GO terms are provided in Supplementary Table 7. Regarding the BP category, positive regulation of smooth muscle cell proliferation, MAPK cascade, peptidyl-serine phosphorylation, and gene expression may be the dominant biological functions of ZSYTP against POI. The MF enrichment revealed that ZSYTP may function through multiple molecular pathways, such as identical protein, enzyme, and protein kinase binding. These results suggest that the therapeutic effects of ZSYTP against POI are most likely based on the regulation of gene and protein expression.

#### 3.3.2 KEGG pathway enrichment analysis

Based on the KEGG PATHWAY database, we identified 119 enriched KEGG pathways shared by ZSYTP and POI, which are collated in Supplementary Table 7. Among the top 20 common KEGG pathways, pathways in cancer, proteoglycans in cancer, and EGFR tyrosine kinase inhibitor resistance, not only accounted for the highest gene ratio but also had the lowest *p*-value scores (Fig. 5B). Moreover, the PI3K-AKT signaling pathway also showed remarkably high enrichment. These cell proliferation- and cell growth-related pathways were closely involved in POI, which might be associated with the mechanisms of ZSYTP in treating POI.

### 3.4 Molecular docking

**Fig. 6:**
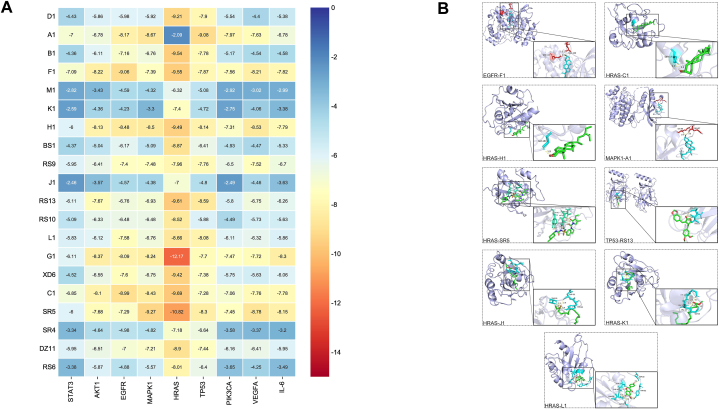
Molecular docking of key target proteins with potential chemical components. (A) The lowest binding energy of compounds-targets docking simulation (kcal/mol). (B) Virtual docking of bioactive ingredients in ZSYTP with critical potential targets.

Molecular docking simulation is a useful method for studying the interaction of molecules and evaluating the accuracy of target predictions. The lowest binding energy of key target proteins to their designated compounds is illustrated in Fig. 6A. According to previous studies, the binding strength of a protein is negatively related to its energy; < −1.2 kcal/mol and < −5.0 kcal/mol can be accredited as practical and dynamite docking, respectively [36]. In terms of potential targets, HRAS and TP53 possessed the best binding outcomes. Among the different ingredients, G1, F1, SR5, and C1 associated relatively more easily compared to other ingredients. The details of nine targets docked to the specific ligands at the lowest binding energy are shown in Fig. 6B, implying considerable affinity between key targets of POI and the major ingredients of ZSYTP.

### 3.5 Effect of ZSYTP on ovarian histology, follicle change, and female hormone level

**Fig. 7:**
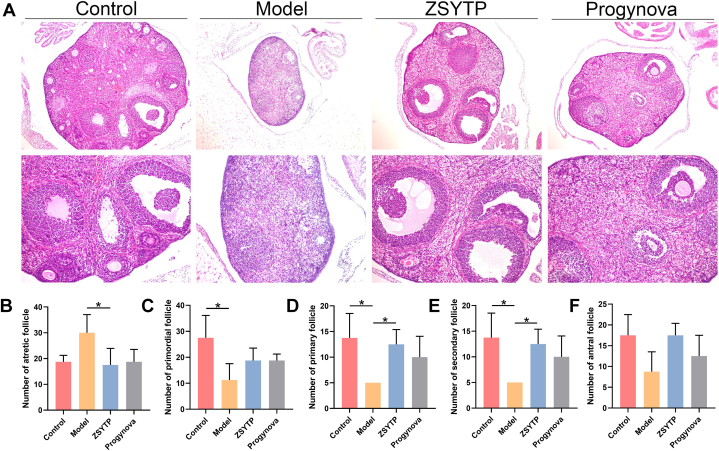
ovarian histopathological and follicle change after ZSYTP treatment. (A) HE staining of the ovary. (B–F) Counting of ovarian follicles in each stage.

**Fig. 8:**
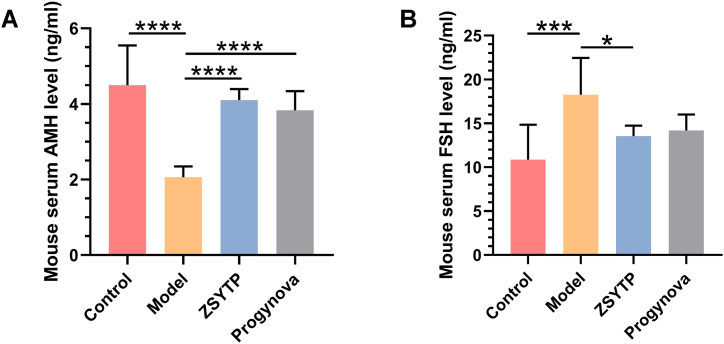
Endocrine changes after ZSYTP treatment. (A) The level of AMH in each group. (B) The level of FSH in each group. *p < 0.05, **p < 0.01, ***p < 0.001, ****p < 0.0001.

As our immunohistochemical observations by H&E staining indicated in our mouse POI models, ZSYTP and Progynova attenuated the histopathological changes in ovaries induced by CTX (Fig. 7A). After exposure to CTX for two weeks, the number of atretic follicles increased significantly, as shown in the model group (*p* < 0.05), while other follicles, except antral follicles, declined markedly compared with the control group (*p* < 0.05; Fig. 7B–F). Moreover, the configuration of the ovarian interstitium in the model group became sparse and disorderly. Fig. 8 shows the levels of POI-related hormones, revealing that short-term exposure to CTX not only enhanced the level of FSH (*p* < 0.001) but also led to a remarkable decrease in the level of AMH (*p* < 0.0001). These results corresponded to diminished ovarian reserves, implying that our construction of a mouse POI model was successful.

The ZSYTP could alleviate the toxic effects in POI mouse models. As shown in Fig. 7, in contrast with the model group, the addition of ZSYTP not only decreased atretic follicles but also significantly increased primary and secondary follicles (*p* < 0.05). We observed no significant difference between the model and Progynova groups. Regarding endocrine regulation, AMH levels in the ZSYTP and Progynova groups were prominently higher than that in the model group (*p* < 0.0001), while FSH levels remained low only in the ZSYTP group (*p* < 0.05; Fig. 8). These results suggested a stronger therapeutic effect of ZSYTP against CTX-induced POI compared to Progynova.

### 3.6 ZSYTP decreases ovarian apoptosis but not via the caspase-3 pathway

**Fig. 9:**
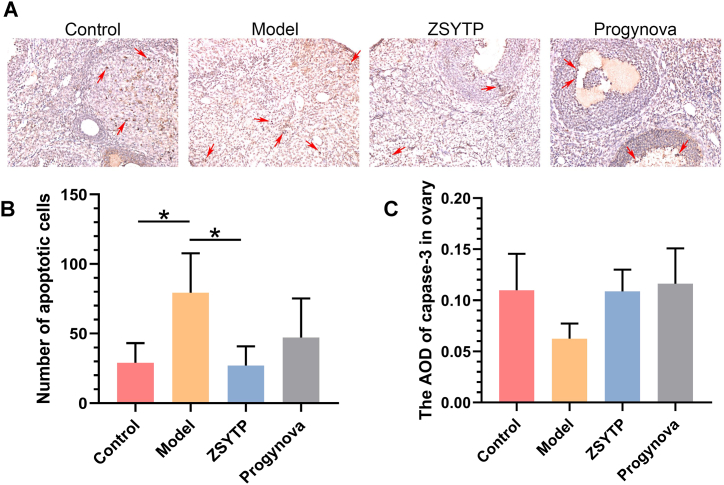
Immunohistochemical analysis of ovarian apoptosis. (A) TUNEL assay of mice ovaries. (B) TUNEL-positive cell count. (C) The average optical density (AOD) of caspase-3. AOD = IntDen/Area. *p < 0.05, **p < 0.01, ***p < 0.001, ****p < 0.0001.

As the core pathogenesis of POI is directly relevant to the abnormal atresia of primordial follicles (PMF) and excessive apoptosis of oocytes, we evaluated the apoptosis of mice ovaries in this study [30]. As shown by the TUNEL assay, the exposure to CTX exerted a measurable effect on the number of apoptotic cells, while ZSYTP significantly ameliorated this effect (*p* < 0.05; Fig. 9A and B).

Caspase-3, which is considered the central executioner protease of apoptosis, is a common downstream effector of multiple apoptotic pathways and consequently instrumental in this process [37]. As evidenced by immunohistochemical staining, we detected no significant difference in caspase-3 expression between all groups, which contradicted the results of previous studies [38,39]. Our current findings therefore suggested that ZSYTP might mediate its inhibitory effects on cell death through other pathways.

### 3.7 Transcriptome analysis

#### 3.7.1 Data description

**Fig. 10:**
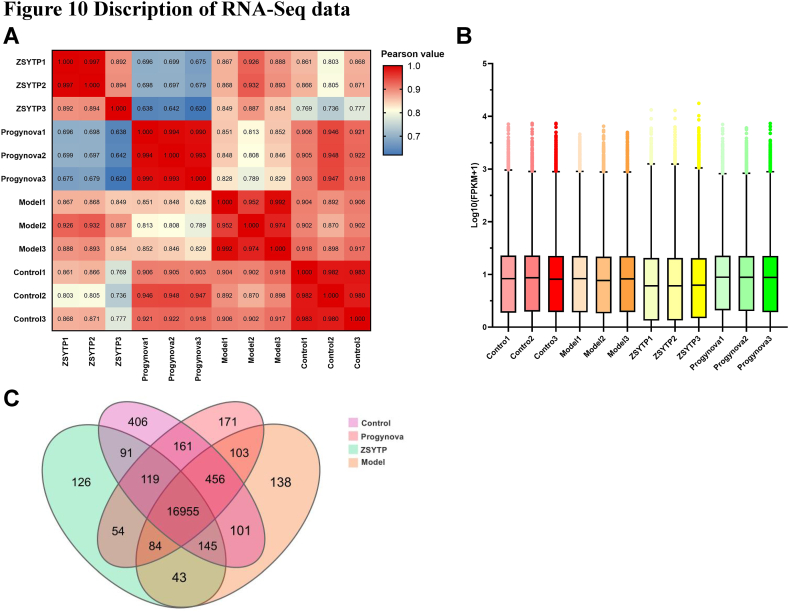
Description of RNA-seq data. (A) Correlation of samples. (B) Gene expression in each sample. (C) Genes expression in each group.

The statistics of the mice RNA sequence data are shown in Table 2. To validate the relevance of gene expression among the different samples, we computed the Pearson correlation coefficients of the total expression level between every two samples. As shown in Fig. 10A, the reproducibility in the same group was notably high, and the within-sample correlations were considerably higher than those between samples. As depicted by the corresponding box plot, the distribution of gene expression levels among different samples was highly consistent (Fig. 10B). These results indicated that our RNA-seq data had a highly satisfactory mapping ratio, reproducibility, and normalization and could ther be used for subsequent analysis.

**Table 2:** Statistics of mice RNA-Seq data.

Sample Name	Total Raw Reads (M)	Total Clean Reads (M)	Total Clean Bases (Gb)	Total Mapping (%)	Uniquely Mapping (%)
Control1	47.19	45.25	6.79	92.82	83.73
Control2	45.8	44.06	6.61	93.55	84.48
Control3	47.19	45.36	6.8	93.24	84.48
Model1	47.19	45.19	6.78	93.08	83.63
Model2	47.19	44.97	6.75	92.48	82.88
Model3	47.19	45.22	6.78	92.8	83.24
ZSYTP1	47.11	45.22	6.78	93.48	84.15
ZSYTP2	47.19	45.26	6.79	93.15	83.59
ZSYTP3	50.79	49.43	7.41	95.29	84.58
Progynova1	45.44	43.89	6.58	93.37	83.3
Progynova2	47.19	45.09	6.76	92.95	83.17
Progynova3	47.19	45.37	6.81	93.12	83.72

The graph of the RNA-seq data concerning the number of expressed genes in each group is shown in Fig. 10C (FPKM >0). A total of 16,955 genes were shared between all groups. The number of uniquely expressed genes was as follows: 406 (control), 138 (model), 126 (ZSYTP), and 171 (Progynova).

#### 3.7.2 Differentially expressed genes and functional analysis

**Fig. 11:**
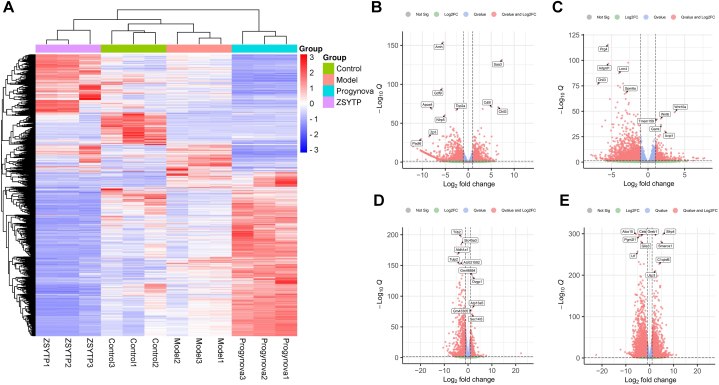
Comparative gene expression between ZSYTP and Progynova. (A) Heatmap of total DEGs expression among different groups. (B) Volcano plot of DEGs between Model and Control group. (C) Volcano plot of DEGs between ZSYTP and Model group. (D) Volcano plot of DEGs between Progynova and Model group. (E) Volcano plot of DEGs between ZSYTP and Progynova group. Q-value <0.05, Fold change >2.

To study how ZSYTP ameliorates CTX-induced POI and to compare its effect with that of Progynova, we performed four sets of differentially expressed gene (DEG) analyses with |log_2_FC| > 1 and Q-value <0.05. The number of DEGs between the control and model groups was 1,779, consisting of 842 significantly upregulated and 937 significantly downregulated genes. Compared with the model group, there were 542 up- and 2172 down-regulated transcripts in the ZSYTP group, while in the Progynova group, we identified 1215 up- and 960 down-regulated transcripts. Notably, there were 5870 DEGs (2030 upregulated and 3840 downregulated) between the ZSYTP and Progynova groups, which accounted for 31.8% of the 18,475 co-expressed genes, suggesting that the pharmacological mechanism of ZSYTP might differ from that of Progynova. Detailed information of the DEGs can be found in sto visualize transcript abundance in each sample for the total of 6939 DEGs (Fig. 11A). The identified DEGs in pairwise comparison are depicted by a volcano plot (Fig. 11B–E).

**Fig. 12:**
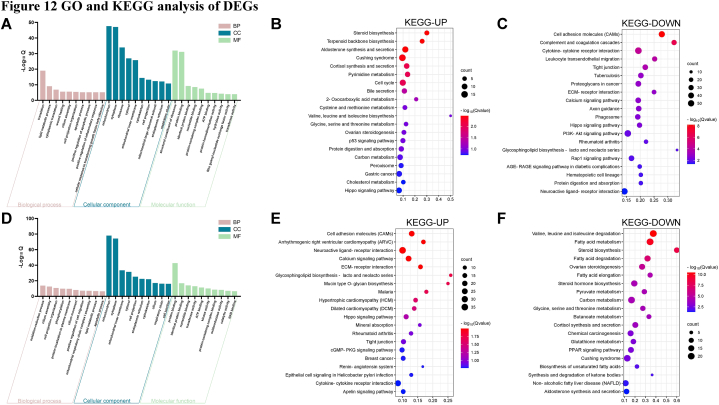
GO and KEGG analysis of DEGs. (A) GO enrichment analysis of DEGs between ZSYTP and Model group. (B) KEGG analysis of upregulated DEGs between ZSYTP and Model group. (C) KEGG analysis of downregulated DEGs between ZSYTP and Model group. (D) GO enrichment analysis of DEGs between Progynova and Model group. (E) KEGG analysis of upregulated DEGs between Progynova and Model group. (F) KEGG analysis of downregulated DEGs between Progynova and Model group.

To elucidate the different mechanisms of action between ZSYTP and Progynova, we conducted GO and KEGG enrichment analyses of the identified DEGs to characterize the predominant signaling pathways. As shown in Fig. 12A, the DEGs between model and ZSYTP groups were involved in multiple functional pathways, including protein translation, lipid metabolism, apoptosis, and the inflammatory response. Pathway enrichment analysis indicated that upregulated DEGs between the ZSYTP and model groups were highly associated with the generation of steroid, aldosterone, and cortisol, while the downregulated DEGs were involved in several inflammation-related and immune-related pathways, including cell adhesion molecules (CAMs), complement and coagulation cascades, cytokine-cytokine receptor interaction, leukocyte transendothelial migration, and ECM-receptor interaction (Fig. 12B and C). Meanwhile, various pathways, including the Hippo and PI3K-AKT signaling pathways, were linked with the regulation of apoptosis or proliferation. These results revealed that ZSYTP might protect against CTX-induced POI mainly by promoting ovarian hormone synthesis, suppressing the inflammatory response, and regulating cell proliferation and apoptosis.

The GO functional analysis of the Progynova vs. control comparison, showed more associations with oxidative phosphorylation and cell junction or migration relative to the analysis of the ZSYTP vs. control group (Fig. 12D). The KEGG pathway enrichment also revealed differences between ZSYTP and Progynova in the mechanism of attenuating CTX-induced POI (Fig. 12E and F). Upregulated DEGs between the Progynova and control groups were significantly enriched in CAMs, ECM-receptor interaction, and mucin-type O-glycan biosynthesis, which had close relations with cell adhesion and migration. The calcium signaling pathway was also highlighted in KEGG-UP. The KEGG-DOWN results of the Progynova vs. control comparison, revealed significant enrichment in pathways related to lipid metabolism and steroid hormone biosynthesis, which indicated inhibition of sex hormone generation.

## 4 Discussion

The prevalence of POI and early menopause is gradually becoming more frequent and currently affects more than 10% of women, which corresponds to an increase compared to previous evaluations [40]. Although there are a variety of novel approaches for treating POI, HRT still remains the preferred therapeutic option, as it can significantly relieve estrogen deficiency-related symptoms, such as hot flushes and insomnia, thereby greatly improving the quality of life for women affected by POI [7]. Nonetheless, the use of hormone replacement may have potential adverse effects on lipid profiles and hemostatic factors, that consequently increase the risk of cardiovascular events such as venous thromboembolism and coronary heart disease [[41], [42], [43]]. Moreover, HRT is also unsuitable for women who have contraindications such as certain hormone-dependent tumors, especially breast and endometrial cancer, as it may enhance their risk of occurrence [[44], [45], [46], [47]]. In this context, it is imperative to develop non-HRT-based interventions for both POI patients and physicians. Chinese herbal medicine, a time-honored traditional form of medicine developed from numerous clinical practices over thousands of years, has provided many candidate herbs and formulations for the treatment of POI [48]. From the perspective of TCM, diminished ovarian reserve (DOR) can be classified into different TCM syndrome types according to their pathogenesis [[49], [50]]. Among these, kidney deficiency is the basic syndrome type of DOR, suggesting an important role of kidneys in ovarian function. In contrast, the spleen is responsible for the generation of blood. These two viscera interact with each other and cooperate to maintain a normal menstrual cycle. Hence, deficiencies in the kidneys and spleen cause menstrual disorders that, in turn, lead to female infertility [51].

Based on this, ZSYTP has been applied to treat POI and has displayed a favorable clinical effect [[9], [12]][52]. Its prescription was first drafted in the early 1960s by Professor Yuankai Luo, to prevent and cure miscarriage and infertility caused by deficiencies of the kidneys and spleen. This formulation consisted of 15 Chinese medicinal components. Of these, dodder seed and ginseng are considered the most important compounds, with the essential properties to replenish kidney function. Japanese teasel, parasite scurrula, eucommia, medicinal Indian mulberry, and degelatined deer-horn are considered as the secondary important compounds, which can benefit kidney function and tocolysis. Pilose asiabell, largehead atractylodes, Chinese wolfberry fruit, adhesive rehmannia cocked root, tuber fleeceflower, and donkey hide gelatin act together as adjunctive drugs to tonify the spleen and kidney and nourish blood production. The conductant drugs, argy wormwood leaf and villous amomum, mimic the pharmacological effect of other drugs by warming the meridian and organs to stop bleeding [53]. The ZSYTP might enhance ovarian function mainly by regulating hormone levels and improving follicle development and ovulation [9,[54], [55], [56]]. While the underlying therapeutic mechanism of ZSYTP against POI has been described in literature [[57], [58], [59], [60]]^-58−60^, a specific therapeutic target of ZSYTP against POI remains undefined.

In this study, we predicted potential targets of ZSYTP against POI using a network pharmacology approach. Although previous studies have performed network pharmacology analysis and concluded that ZSYTP could be ameliorating POI via glucose metabolism and the HIF-1 signaling pathway, these results were not supported by sufficient experimental validation [36]. In addition, the use of different pharmacological network analysis methods and databases would inevitably lead to different conclusions. In our network prediction, quercetin, kaempferol, and mandenol were identified as the major bioactive components of ZSYTP, while PIK3CA, MAPK1, and AKT1 were presumed as the potential therapeutic targets of POI. We therefore hypothesized that ZSYTP might mitigate POI through multiple KEGG-identified pathways, including the PI3K-AKT, AGE-RAGE, HIF-1. Molecular docking preliminarily validates the accuracy of our network predictions, proving the main main components in ZSYTP can bind closely to nine target proteins, thereby playing a biological role.

To compare the difference between ZSYTP and traditional HRT, we constructed a mouse model of POI by intraperitoneal injection with CTX, followed by administration of ZSYTP or Progynova. Although a commonly used drug for chemotherapy and immunosuppressive therapy, CTX can cause ovary damage and is widely employed to establish POI animal models. In regard to an analysis of published data, exposure to CTX had definite adverse effects on both the PMF pool and growing follicles, increasing follicular atresia and apoptosis in ovaries [35]. The pathological changes observed in our POI model conform with previous findings. Luan et al. also found that administration of CTX resulted in the loss of primordial oocytes through apoptotic pathways [61]. Although our results indicated an upregulation in apoptosis after CTX exposure, there seemed to be no significant correlation with the caspase-3 pathway, which differs from the observations of other studies [[39], [62]]. We surmise that this disparity might be ascribed to the method of determining caspase-3, as another study also found that cleaved caspase-3 staining can only be observed in primary, secondary, and pre-antral follicles (except PMF) [38]. Moreover, a reasonable hypothesis about why the mRNA levels of apoptosis-related molecules were similar between the Model and ZSYTP groups might be because ZSYTP mainly regulates the phosphorylation of the PI3K-AKT pathway members post-translationally, and not their transcription. Another possible explanation might concern the non-apoptotic effects of caspase-3 on cell survival, proliferation, and differentiation, which seems contradictory to its well-known function as a crucial inducer of apoptosis [63]. To thoroughly clarify this potential discrepancy, further studies are warranted.

Histopathological observation highlighted a definitive improvement in the POI model after treatment with ZSYTP, which slightly outperformed Progynova in terms of treatment results. To thoroughly detect changes in gene expression among each group, we performed transcriptome sequencing. Analysis of DEGs demonstrated the reliability of our network pharmacological analysis, revealing that a group of similar target genes may be involved in the treatment of POI using ZSYTP. Notably, the gene expression pattern of the Progynova group was rather different from that of the ZSYTP group, implying that these two drugs have diverse pharmacological mechanisms. We posited that ZSYTP might counter the CTX-induced ovarian histopathological damage and hypothalamus-pituitary-gonadal axis disorder mainly via the PI3K-Akt, Hippo, Rap1, and AGE-RAGE signaling pathways, which are implicated in cell proliferation, apoptosis, migration, lipid metabolism, immune response, and inflammation.

The PI3K-AKT signaling pathway is crucial to mammals, covering a wide range of cellular processes, including cell growth, proliferation, apoptosis, and metabolism [64]. In addition, this pathway also plays a role in the DNA damage response, PMF activation, and ovarian aging [65]. By regulating the PI3K-AKT signaling pathway, ovarian function can be effectively restored following POI [[66], [67], [68]]. It has been observed that exposure to CTX will over activate PMFs, leading to follicular depletion and POI, while suppression of the PI3K/Akt/mTOR and the PI3K/Akt/Foxo3a pathways can prevent CTX-induced ovarian reserve loss by both, inhibiting PMF activation and reducing apoptosis [[69], [70], [71]]. Transcriptome analysis showed several inhibitors of PMF, the expression of PTEN, p27Kip1, Foxl2 and mTORC1 were remarkably recovered after treating with ZSYTP, revealing that ZSYTP may resist POI by reducing PMF hyperactivation.

HIF-1 signaling pathway is another downstream pathway regulated both by PI3K-AKT and mTOR signaling pathway. Its core element HIF-1, which can be activated under oxygen deprivation and upregulate various downstream targets, including VEGF, to compensate for the low oxygen stress, consist of two protein subunit, HIF-1α and HIF-1β [72]. HIF-1α is the active submit, accumulated in cytoplasm under anaerobic environment and mediates hypoxic adaptive response. Li et al. reported that suppressing HIF-1α activity blocked ovulation and caused atresia in large follicles [73]. As oxygen regulation plays a critical role in follicle development and corpus luteum formation, any disorder during a hypoxia-induced response might lower female fertility, leading to polycystic ovary syndrome or POI [74]. Many studies have indicated the possibility of recovering ovarian function by regulating HIF-related pathways [[75], [76], [77]]. Among these, HIF-1α/BNIP3/Beclin1 is the predominant signaling pathway. BNIP3 is a proapoptotic protein belonging to BCL-2 family, playing an important role in hypoxia-induced autophagy [78]. BECN1 gene encodes Beclin1 protein, a direct executor of autophagy. When the expression of BNIP3 increases, the Bcl-2-Beclin1 complex disrupted and Beclin1 released, mediating other autophagy-related protein located in phagosome thus regulates the formation and maturation of autophagosome. Revealed by RNA Seq, the expression of HIF-1α in ovary was decreased by CTX exposure (log2FC −0.8727, Q-value 0.0007). After drug intervention, the mRNA level of HIF-1α was augmented significantly in Progynova group (log2FC 0.53, Q-value 5.7284e-9), while not in ZSYTP group (Q-value 0.59). Interestingly, the expression of BNIP3 (log2FC 0.36, Q-value 0.02) and BECN 1 (log2FC 0.31, Q-value 0.02), was slightly enhanced in ZSYTP group compare to Model group, while in Progynova group was both downregulated (log2FC −0.67, Q-value 2e-8) (log2FC −0.34, Q-value 7.49e-4). One probable conjecture is ZSYTP regulate autophagy in ovary via other signaling pathway, such as AMPK and p53 pathway.

The AGE-RAGE signaling axis is a well-studied cascade related to a diverse range of diseases, especially diabetes mellitus, and it activates various downstream effector pathways, such as the PI3K/AKT, MAPK/ERK, and NADPH oxidase signaling cascades, leading to the production of pro-inflammatory cytokines and reactive oxygen species (ROS), consequently resulting in cell damage in AGE-related diseases [79]. Furthermore, the accumulation of AGEs in ovarian follicles might elicit early ovarian aging and regulate follicular growth [80]. Many phytochemicals, including quercetin and iridoids, can strongly inhibit the AGE–RAGE axis signaling [81]. These phytochemical compounds are enriched in several herbs contained in ZSYTP and likely produce a considerable protective effect against POI. KEGG enrichment analysis of DEGs between Model and ZSYTP group showed that although the mRNA level of RAGE has no significant change, ZSYTP downregulated a group of molecules in the downstream of AGE-RAGE signaling pathway, involving TGF-β, PI3K-AKT and JAK-STAT signaling pathway. Our finding is consistent with previous conclusion.

However, several discrepancies were present between our bioinformatic prediction and experimental results. For instance, in the network analysis, HRAS and STAT3 were predicted as hub genes between ZSYTP and POI, and HIF-1 and VEGF signaling pathways were highly enriched, whereas RNA-seq suggested that they might have no significant function in ZSYTP against POI. One plausible explanation might be that when performing the PPI network analysis of the overlapping genes between ZSYTP and POI, we failed to consider whether these potential target genes were truly abundant in the drug or disease groups. Therefore, certain target genes of marginal compounds in ZSYTP might be highlighted, and vice versa. To minimize this deviation, we performed concurrent component-target network analyses. However, biases still existed between the prediction of pharmacological networks and the actual results, and therefore, certain conclusions drawn from the bioinformatics analysis may not be completely reliable. This further underscores the importance for reasonable follow-up experimental validation of computational predictions.

This research had several limits. We used only RNA-seq to quantify gene expression without using other methods, such as PCR, to further verify the confidence level of the sequencing results. In the near future, we are planning to perform complementary Western blot and qPCR assays to further clarify the controversial results between network prediction and RNA-Seq during a follow-up study. Another potential pitfall was the limited number of mice used, which, to a certain extent, might have affected the authenticity of our conclusions. As different chemicals might induce different kinds of POI, we intend to compare the therapeutic effect of ZSYTP using a diverse range of POI-inducers in a future study. Overall, more basic research is needed to further understand how ZSYTP functions to treat POI and to promote TCM globally.

## 5 Conclusion

In our current research, we predicted the major target genes and pathways of ZSYTP against POI, which were consistent to an extent with the results of previous studies^36 77^. Moreover, our work illustrated how ZSYTP ameliorates POI damage at the mRNA level based on RNA-seq. Based on the sequencing data, we demonstrated that ZSYTP could mediate anti-POI effects by downregulating PI3K-AKT, Hippo, AGE-RAGE, and Rap1 signaling pathways and inflammation, while concomitantly upregulating steroid biosynthesis, which was overall a markedly different mode of action from Progynova. These findings not only confirm the hypotheses presented in other studies but also contest several established views on the molecular mechanism of ZSYTP against POI, such as the HIF-1 signaling cascade. Based on these results, we believe that ZSYTP would be an excellent drug candidate as a complementary and alternative medicine for treating POI, while intending to investigate this in greater detail in the future.

## Author contribution statement

Jia Hu: Conceived and designed the experiments; Wrote the paper; Performed the experiments; Analyzed and interpreted the data. </p>

Zifan Song: Performed the experiments; Analyzed and interpreted the data; Wrote the paper. </p>

Kuangyu Song, Hongru Zhao: Performed the experiments; Analyzed and interpreted the data. </p>

Yuanqiao He: Performed the experiments. </p>

## Data availability statement

Data will be made available on request.

## Author contributions

JH conceived and designed the experiments. JH, ZS, KS, HZ and QH performed the experiments. ZS, KS, HZ and JH analyzed and interpreted the data.ZS and JH wrote the paper. All authors contributed to the article and approved the submitted version.

## Declaration of competing interest

The authors declare that they have no known competing financial interests or personal relationships that could have appeared to influence the work reported in this paper.

## Glossary

AGE: Advanced glycation end
ANOVA: Analysis of variance
AOD: Average optical density
BP: Biological process
CAM: Cell adhesion molecules
CC: Cellular component
DEG: Differentially expressed genes
DOR: Diminished ovarian reserve
ESHRE: European Society of Human Reproduction and Embryology
FSH: Follicle Stimulating Hormone
GO: Gene Ontology
HRT: Hormone replacement therapy
KEGG: Kyoto Encyclopedia of Genes and Genomes
MF: Molecular function
OD: Optical density
PMF: Primordial follicles
POF: Premature ovarian failure
POI: Premature ovarian insufficiency
PPI: Protein interaction
ROS: Reactive oxygen species
SD: Standard deviations
TCM: Traditional Chinese medicine
TUNEL: Transferase dUTP nick end-labeling
