# Supplementary figures and images for: Network analysis and experimental approach to investigate the potential therapeutic mechanism of zishen yutai pills on premature ovarian insufficiency
Source: Heliyon. 2023 Sep 17;9(9):e20025. doi: 10.1016/j.heliyon.2023.e20025 (PMC10559743; doi:10.1016/j.heliyon.2023.e20025)

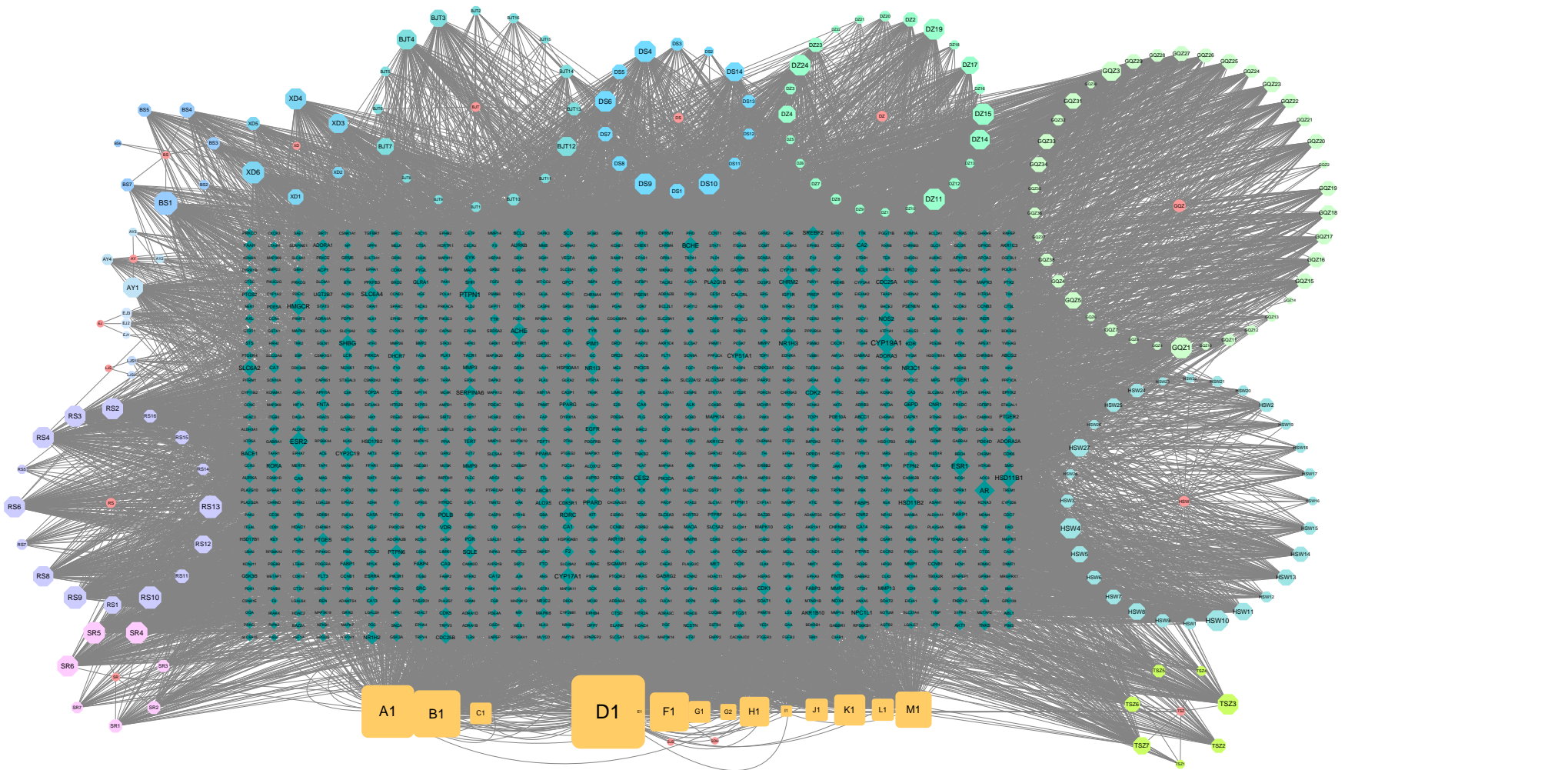

Supplement: Multimedia component 9 [file mmc9.pdf]
